# Stereotactic body radiation therapy for oligometastatic pulmonary tumors from cervical cancer

**DOI:** 10.1111/ajco.13159

**Published:** 2019-05-08

**Authors:** Xiaorong Hou, Weiping Wang, Fuquan Zhang, Ke Hu

**Affiliations:** ^1^ Department of Radiation Oncology Peking Union Medical College Hospital. Chinese Academy of Medical Sciences & Peking Union Medical College No. 1 Shuaifuyuan, Wangfujing Dongcheng District Beijing China

**Keywords:** cervical cancer, pulmonary metastases, radiotherapy, SBRT

## Abstract

**Objective:**

To evaluate the efficacy and toxicity of stereotactic body radiation therapy (SBRT) for oligometastatic pulmonary tumors from cervical cancer.

**Methods:**

A total of 29 oligometastatic pulmonary lesions from cervical cancer in 19 patients were treated with SBRT in our institute from 2011 to 2016. Thirteen patients (68.4%) suffered with solitary lung metastasis, three patients (15.8%) with multiple unilateral lesions and three patients (15.8%) with bilateral lesions. The median size of lung lesions was 2 cm (0.7–5.6 cm). Patients underwent cone‐beam CT before the delivery of SBRT. The most common dose fractionation schemes were 64 Gy in eight fractions (eight lesions) and 56 Gy in seven fractions (seven lesions). Nine patients (47.4%) received systemic chemotherapy.

**Results:**

The median follow‐up was 9.5 months (3.0–62.4 months). The one‐year overall survival (OS), progression‐free survival (PFS) and local control (LC) rates were 76.8%, 55.8% and 75.6%, respectively. The median PFS was 12.7 months. Six patients (31.6%) gained more than 20 months disease‐free survival. Eleven patients (57.9%) experienced tumor relapse, including seven patients with pulmonary relapse and four patients with extra‐pulmonary disease. Only one patient (5.3%) experienced symptomatic radiation pneumonitis (grade 2).

**Conclusion:**

SBRT was an efficacy and safe approach for patients with oligometastatic pulmonary tumor from cervical cancer. SBRT should be considered as a potential alternative to resection for these patients.

## 1 INTRODUCTION

Cervical cancer is one of the most common cancers in China. It was estimated that there were 98.9 thousand new cervical cancer cases and 30.5 thousands deaths in 2015.^1^ With the changes of treatment pattern and development of treatment technique, the local control (LC) of cervical cancer improved significantly. After definitive treatment, there was more distant failure than local failure.^2^, ^3^ Pulmonary metastasis is one of the most common sites of distant failure. It was reported that the incidences of pulmonary metastasis were 1.8–2.1%.^4^, ^5^

For pulmonary metastases from cervical cancer after initial treatment (radical surgery or definitive radiotherapy), pulmonary resection is an efficacy approach, with a 5‐year survival of more than 30%.^6^, ^7^ However, only patients with few metastatic lesions limited to the lungs and good general condition could receive pulmonary resection and only a small number of patients meets these criteria. Most patients with pulmonary metastases from cervical cancer were treated with chemotherapy or radiotherapy. As a palliative treatment, the survival after single chemotherapy was poor. Panek et al. reported that, after platinum‐5‐fluorouracil chemotherapy, only 12% of patients with pulmonary metastases from cervical cancer achieved complete response (CR). The overall survival (OS) and progression‐free survival (PFS) at 3 years were 17.6% and 14.3%, respectively.^8^ Patients with oligo‐pulmonary metastases from cervical cancer still have opportunity to have long‐term survival. Therefore, just giving these patients single chemotherapy is unreasonable.

In the past, radiotherapy was often used as a palliative approach for pulmonary metastases. As a new technique, stereotactic body radiation therapy (SBRT) could accurately deliver high dose to the lesions in few fractions. In pulmonary metastasis from rectum and soft tissue sarcoma, SBRT had been proved to be a safe and effective approach^9^, ^10^, ^11^ and became an important alternative to resection.

For the use of SBRT in pulmonary metastases from cervical cancer, researches are limited. In this article, we retrospectively analyzed the efficacy and toxicity of SBRT for patients with pulmonary metastases from cervical cancer after definitive treatment in our institute.

## 2 MATERIALS AND METHODS

### 2.1 Patients

We retrospectively analyzed patients with pulmonary metastases from cervical cancer treated with SBRT from January 2011 to July 2016 in Peking Union Medical College Hospital. The inclusive criteria were as follows: biopsy‐diagnosed cervical cancer; received definitive treatment for cervical cancer (surgery or radiotherapy) and achieved clinical CR after treatment; diagnosed of pulmonary metastases after a period of disease‐free survival (DFS); pulmonary metastases were treated with SBRT; KPS score ≥60. The exclusive criteria included: with extra‐pulmonary lesions before or simultaneous with pulmonary metastases; pulmonary metastases treated with radiotherapy or surgery before SBRT. There were 19 patients eligible for this research.

The patients, tumor and treatment characteristics were shown in Table 1. The most common histology was squamous cell carcinoma (17 patients, 89.5%). When diagnosed of cervical cancer, the most common stage was stage IB (six patients, 31.6%) and stage IIB (six patients, 31.6%). Fifteen patients (78.9%) received definitive concurrent chemoradiotherapy (CCRT) or radiotherapy as primary treatment and four patients (21.1%) received surgery.

**Table 1:** Patient, tumor and treatment characteristics

Characteristics	Number	Percentage (%)
Histology		
SCC	17	89.5
Adenocarcinoma	1	5.3
Neuroendocrine carcinoma	1	5.3
Primary FIGO stage		
IB	6	31.6
IIA	3	15.8
IIB	6	31.6
IIIB	4	21.1
Primary treatment approach		
Concurrent chemoradiotherapy	14	73.7
Radiotherapy	1	5.3
surgery	2	10.5
Surgery+radiotherapy	2	10.5
DFI		
≤12 months	12	63.2
>12 months	7	36.8
SCC Antigen		
<1.5 ng/mL	9	47.4
1.5–10 ng/mL	5	26.3
>10 ng/mL	5	26.3
No. of pulmonary metastases		
1	13	68.4
2	3	15.8
3	2	10.5
4	1	5.3
Unilateral or bilateral lung metastases		
Unilateral lung metastases	16	84.2
Bilateral lung metastases	3	15.8
Diameter of lung metastases		
<1 cm	4	13.8
1–1.9 cm	10	34.5
2–2.9 cm	8	27.6
≥3 cm	7	24.1
Location of lung metastases		
Central	8	27.6
Peripheral	21	72.4
Fractionated dose (BED)		
64 Gy in 8 fractions (115.2 Gy)	8	27.6
56 Gy in 7 fractions (100.8 Gy)	7	24.1
48 Gy in 6 fractions (86.4 Gy)	4	13.8
60 Gy in 10 fractions (96.0 Gy)	2	6.9
50 Gy in 10 fractions (75.0 Gy)	2	6.9
60 Gy in 6 fractions (120.0 Gy)	1	3.4
60 Gy in 15 fractions (84.0 Gy)	1	3.4
50 Gy in 5 fractions (100.0 Gy)	1	3.4
48 Gy in 4 fractions (105.6 Gy)	1	3.4
45 Gy in 3 fractions (112.5 Gy)	1	3.4
40 Gy in 10 fractions (56.0 Gy)	1	3.4
Chemotherapy		
Yes	9	47.4
No	10	52.6

Abbreviations: BED, biological effective dose; SCC, squamous cell carcinoma.

At the time of SBRT, the median age was 47 years old (range, 26–67). The time interval from CR of primary tumor to the appearance of lung metastases was disease‐free interval (DFI). The median DFI was 10.7 months.

There were 29 lesions for these 19 patients in total. The mean number of lung metastases was 1.5 (range, 1–4). Thirteen patients (68.4%) had single metastases, three patients (15.8%) suffered with multiple lung metastases in single lung (two patients with two lesions and one patient with three lesions) and three patients had bilateral lung lesions (one patient with two lesions, one patient with three lesions, one patient with four lesions). The median diameter of the lesions was 2 cm (range, 0.7–5.6 cm).

### 2.2 Treatment

A CT simulation was performed with 16‐slice Philips Brilliance Big Bore CT at a slice thickness of 5 mm, and patients were immobilized with thermoplastic. The gross tumor volume (GTV) was contoured on the CT image for each patient. The planning target volume (PTV) was defined as the GTV plus a 5–10 mm margin. The prescribed dose was decided considering the size and location of the tumors.

Coplanar fixed‐field intensity modulated radiation therapy plan was made and treatment was delivered with a Varian Trilogy linear accelerator (Varian Medical Systems, USA). A cone beam CT (CBCT) was acquired and registered to the planning CT before every treatment. The target position error was corrected by shifting the treatment couch. The prescription dose was modulated according to size, number and location of the lesions. The most common prescribed doses included 64 Gy in eight fractions (eight lesions) and 56 Gy in seven fractions (seven lesions). The median fractions of SBRT was seven fractions (range 3–15 fractions). The fractionated dose ranged from 4 to 15 Gy and the most common one was 8 Gy (19 lesions, 65.5%). We calculate the biological effective dose (BED, *α*/*β* = 10 for tumors) for every tumor. The median BED was 100.8 Gy (range 56–120). Of the 29 pulmonary metastases, the BED of 18 lesions (62%) was higher than 100 Gy and the BED of 26 lesions (89.7%) was higher than 80 Gy.

Nine patients (47.4%) received chemotherapy. The chemotherapy regime included paclitaxel and carboplatin (five patients), paclitaxel and cisplatin (two patients), cisplatin and 5‐Fu (one patient), cisplatin alone (one patient). The median courses of chemotherapy were four courses (range 3–6). Chemotherapy was performed before radiotherapy in three patients and after radiotherapy in six patients. The median interval between radiotherapy and chemotherapy was 1.5 months (range, 0.4–3.1 months). The detailed treatment characteristics were shown in Table 1.

### 2.3 Outcome evaluation and statistical analysis

Response Evaluation Criteria in Solid Tumors criteria was used to assess the treatment efficacy. Treatment‐related adverse effects were graded according to Common Terminology Criteria for Adverse Effects version 3.0. Statistical analysis was performed using the SPSS version 19.0. OS, PFS and LC were calculated using Kaplan–Meier analysis from the time of SBRT completion. LC was defined as no progression in their radiation field during follow up.

## 3 RESULTS

The median follow‐up was 9.5 months (range, 3.0–62.4). The median follow‐up for patients alive was 18.9 months (range, 3.6–62.4). The 1‐year OS, PFS and LC were 76.8%, 55.8% and 75.6%, respectively. The estimated 2‐year OS, PFS and LC were 76.8%, 37.2% and 75.6%. The median PFS was 12.7 months.

By the end of follow‐up, 11 patients (57.9%) experienced progressive disease. Seven of them had progression in the lung, including two patients with progression in the irradiation field and five patients with new lesions outside the irradiation volume. Four patients had extrapulmonary metastases, including two patients with http://mediastinum lymph nodes metastases, one patient with bone metastases and one patient with liver metastases.

**Figure 1:**
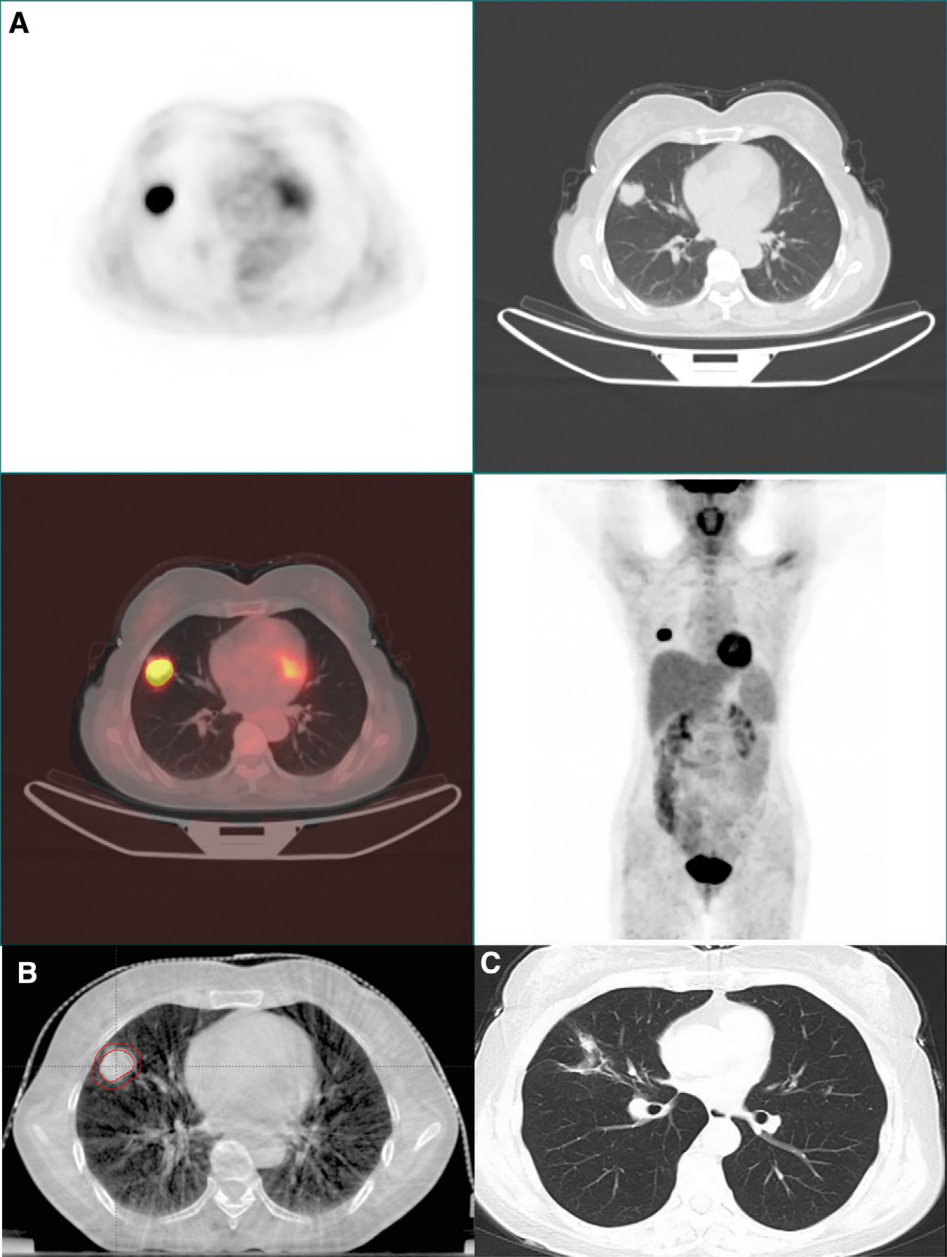
The pretreatment PET/CT images (A), the cone beam CT image before first delivery (B) and the chest CT 40 months after treatment (C) [Color figure can be viewed at http://wileyonlinelibrary.com]

Of the eight patients (42.1%) without progression during follow‐up, six patients (31.6%) appeared to be disease free. Five of these six disease‐free patients had solitary lesion in the lung and the other one patient had three lesions in the bilateral lungs. The median diameter of the lesions was 2.3 cm (range, 1–3.5 cm). The dose fractionations delivered to the tumor included 64 Gy in eight fractions (three lesions), 56 Gy in seven fractions (two lesions), 60 Gy in six fractions (one lesion), 50 Gy in five fractions (one lesion) and 45 Gy in three fractions (one lesion). The median follow‐up of theses six patients was 40.4 months (range, 20.5–62.4). Figure 1 was the PET/CT images before treatment, CBCT image before the first delivery and the CT image 40 months after treatment of a patient with long‐time survival. She was a 47‐year‐old women who diagnosed with cervical squamous cell carcinoma of stage FIGO IIB in November 2009. She was treated with CCRT between December 2009 and February 2010 and acquired clinical CR. In November 2012, the squamous cell carcinoma antigen raised to 3.9 ng/mL. PET/CT scan showed a 2.6‐cm hypermetabolic lesion in the right lung and no evidence of extrapulmonary metastases. The lesion in the lung was treated with SBRT, 56 Gy in seven fractions. The patient acquired CR and DFS until the last follow up (April 2016).

**Figure 2:**
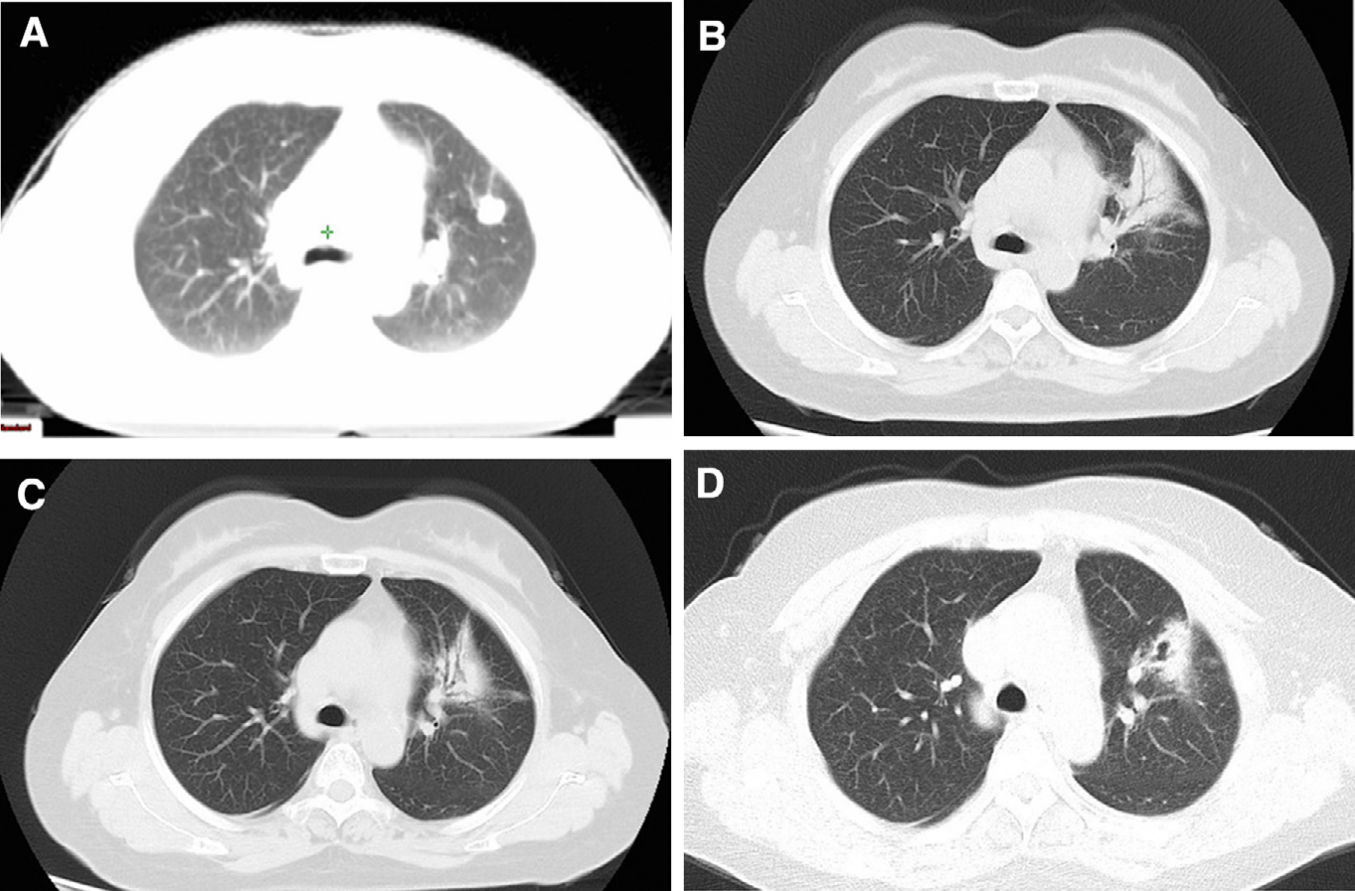
The chest CT images of the patient with grade 2 radiation induced pneumonitis (A: pretreatment image; B: 2 months after radiotherapy; C: 8 months after radiotherapy; D: 22 months after treatment) [Color figure can be viewed at http://wileyonlinelibrary.com]

Symptomatic radiation pneumonitis was observed in one patient (5.3%). This patient received 64 Gy in eight fractions irradiation to 1.5 cm lesion in the left lung and suffered with Grade 2 radiation pneumonitis 2 months later. The symptoms included cough, expectoration and dyspnea. These symptoms limited instrumental activities of daily living but she did not need oxygen uptake. The chest CT images shown large patchy shadow in the left lung. After treated with glucocorticoid, the symptom relieved. The chest CT images before SBRT, at the time of radiation pneumonitis and after the treatment of glucocorticoid were shown in Figure 2.

## 4 DISCUSSION

For oligometastatic pulmonary tumors from cervical cancer, surgery is an important treatment approach. In the study of Anderson et al., six patients with pulmonary metastases from cervical cancer were treated with pulmonary resection. The median survival was 36 months.^5^ Yamamoto et al. reported 29 patients with pulmonary metastases from cervical cancer, which were detected after a disease‐free period after initial treatment and were resected with the intention to cure. A solitary lesion was found in 17 patients, 2 lesions were found in 6 patients, 3 lesions in 3 patients and 4 lesions in 3 patients. The 5‐year DFS rate was 32.9% for all patients. For 23 patients with one to two pulmonary lesions, the 5‐year DFS was 42.2%.^6^ Anraku et al. treated 76 patients with pulmonary metastases from cervical cancer with surgical resection, the 5‐year OS was 45.7%.^7^ For patients with pulmonary metastases from cervical cancer after radical treatment, surgical resection is an efficacy approach. The 5‐year survival was higher than 30%.

For patients with pulmonary metastases, SBRT was an effective treatment option. The 2 or 3‐year LC rate was higher than 70%.^9^, ^10^, ^11^ In the pooled analysis of Germen working group, 700 patients with medically inoperable lung metastases in 20 centers were treated with SBRT. The most common primary tumors included non–small cell lung cancer (NSCLC, *n* = 210), colorectal cancer (*n* = 153), sarcoma (*n* = 51) and so on. Solitary pulmonary metastases were found in 246 patients (42.4%). The median diameter of the lesions was 2.2 cm (range 0.4–9.4 cm). The median single fraction dose of PTV was 12.5 Gy (range 3–33 Gy) and the median number of SBRT fractions was 3 (range, 1–13). The median follow‐up was 14.3 months. The 2‐year LC and OS rates were 81.2% and 54.4%, respectively.^9^ A study from South Korea involved 50 patients (79 pulmonary lesions) with one to three lung metastases from colorectal cancer. A total dose of 40–60 Gy (median, 48 Gy) in three or four fractions was prescribed to the pulmonary lesions with SBRT. The 3‐year OS, PFS and LC rates were 64.0%, 24.0% and 70.6%, respectively.^10^ In the study of Baumann et al., 30 sarcoma patients with 39 pulmonary metastases received 50 Gy in four to five fractions with SBRT. Two‐year OS and LC rates were 43% and 86%, respectively.^11^ In our study, the 2‐year OS, PFS and LC rates were 76.8%, 37.2% and 75.6%, respectively. To our knowledge, this was the first study on SBRT for pulmonary metastases from cervical cancer.

SBRT or stereotactic ablative radiotherapy (SABR) have become an important treatment option for patients with inoperable stage I NSCLC. A pooled analysis indicated that, for operable stage I NSCLC, patients treated with SABR could get better OS compared with lobectomy.^12^ For patients with single or oligo‐pulmonary metastases, SBRT was often used as an treatment option for inoperable tumors or patients refusing surgery, and there was no randomized control trial to compare the efficacy of SBRT and surgery. Yu et al. retrospectively analyzed 58 patients with pulmonary metastases from osteosarcoma. Of them, 27 patients were treated with stereotactic radiosurgery (SRS). A dose of 50 Gy in 10 fractions was delivered to PTV and 70 Gy in 10 fractions was prescribed to the GTV. The other 31 patients were treated by surgical resection. For SRS group and surgical group, the 2‐year OS rates were 40.7% and 48.3% (*P* > 0.05), the 2‐year PFS rates were 33.3% and 38.7% (*P* > 0.05).^13^ In this study, the 2‐year PFS rate was 37.2%. Six of 19 patients (31.6%) appeared to be disease free for more than 20 months. The outcome was close to the data of pulmonary resection.^6^, ^7^ This indicated that, for operable patients, SBRT was also a potential alternative to resection for oligometastatic pulmonary tumors from cervical cancer. Considering the small number of patients and short follow‐up period, we need more studies on this.

According to National Comprehensive Cancer Network (NCCN) guidelines of NSCLC, the dose of SBRT delivered to stage I NSCLC was decided based on the size, location of the tumor. The fractionated dose range from 25 to 34 Gy in one fraction to 60–70 Gy in 8–10 fractions.^14^ For pulmonary metastases, studies on the dose of SBRT were limited. In the previous studies, the dose fractionation delivered included 48 Gy in four fractions or 50–60 Gy in five to eight fractions,^15^ 50 Gy in four to five fractions,^11^ 40–60 Gy(median 48 Gy) in three to four fractions,^10^ median PTV single dose of 12.5 Gy (range 3.0–33.0 Gy) in a median number of three fractions (range 1–13).^9^ The radiation sensitivity of lung metastases varies with different primary tumors. The radiation sensitivity of cervical cancer was comparatively higher than sarcoma, colorectal cancer, renal cell carcinoma and NSCLC et al. In this study, the most common dose fractionations were 64 Gy in 8 fractions (8 lesions), 56 Gy in 7 fractions (7 lesions). The median BED was 100.8 Gy (56–120 Gy). With these dose fractionations, SBRT achieved excellent LC and promising OS. This may provide an acceptable dose fractionation option for pulmonary metastases from cervical cancer.

It was reported that the incidence rates of grade 2 or greater radiation pneumonitis were 0–6.5% for patients with pulmonary metastases when they treated with SBRT.^9^, ^10^, ^11^, ^15^, ^16^ In this study, only 1 (5.3%) patients had grade 2 radiation pneumonitis, which was similar with previous reports.

There were some limitations in this study. The number of patients with pulmonary metastases from cervical cancer was small and the follow‐up period was short. As pulmonary metastases from cervical cancer are comparatively rare,^4^ multi‐institute studies are needed to collect more patients in the future.

SBRT was an efficacy and safe approach for patents with oligometastatic pulmonary tumor from cervical cancer. SBRT should be considered as a potential alternative to resection for these patients.

## CONFLICTS OF INTEREST

The authors declare that they have no conflict of interest.

## FUNDING

This study is supported by the Ministry of Science and Technology of the People's Republic of China (Grant No 2016YFC0105207).

## References

[1] Chen W , Zheng R , Baade PD , et al. Cancer statistics in China, 2015. CA Cancer J Clin. 2016;66:115–132. 26808342 10.3322/caac.21338

[2] Ribeiro I , Janssen H , De Brabandere M , et al. Long term experience with 3D image guided brachytherapy and clinical outcome in cervical cancer patients. Radiother Oncol. 2016;120:447–454. 27157510 10.1016/j.radonc.2016.04.016

[3] Kato S , Ohno T , Thephamongkhol K , et al. Long‐term follow‐up results of a multi‐institutional phase 2 study of concurrent chemoradiation therapy for locally advanced cervical cancer in east and southeast Asia. Int J Radiat Oncol Biol Phys. 2013;87:100–105. 23920390 10.1016/j.ijrobp.2013.04.053

[4] Gunasekera PC , Fernando RJ , Abeykoon SC . Pulmonary metastases from cervical cancer in Sri Lanka. J Obstet Gynaecol. 1999;19:65–68. 15512227 10.1080/01443619966010

[5] Anderson TM , McMahon JJ , Nwogu CE , et al. Pulmonary resection in metastatic uterine and cervical malignancies. Gynecol Oncol. 2001;83:472–476. 11733957 10.1006/gyno.2001.6427

[6] Yamamoto K , Yoshikawa H , Shiromizu K , et al. Pulmonary metastasectomy for uterine cervical cancer: A multivariate analysis. Ann Thorac Surg. 2004;77:1179–1182. 15063230 10.1016/j.athoracsur.2003.06.023

[7] Anraku M , Yokoi K , Nakagawa K , et al. Pulmonary metastases from uterine malignancies: Results of surgical resection in 133 patients. J Thorac Cardiovasc Surg. 2004;127:1107–1112. 15052209 10.1016/j.jtcvs.2003.10.011

[8] Panek G , Gawrychowski K , Sobiczewski P , et al. Results of chemotherapy for pulmonary metastases of carcinoma of the cervix in patients after primary surgical and radiotherapeutic management. Int J Gynecol Cancer. 2007;17:1056–1061. 17466044 10.1111/j.1525-1438.2007.00879.x

[9] Rieber J , Streblow J , Uhlmann L , et al. Stereotactic body radiotherapy (SBRT) for medically inoperable lung metastases‐A pooled analysis of the German working group “stereotactic radiotherapy”. Lung Cancer. 2016;97:51–58. 27237028 10.1016/j.lungcan.2016.04.012

[10] Jung J , Song SY , Kim JH , et al. Clinical efficacy of stereotactic ablative radiotherapy for lung metastases arising from colorectal cancer. Radiat Oncol. 2015;10:238. 26588896 10.1186/s13014-015-0546-x PMC4654895

[11] Baumann BC , Nagda SN , Kolker JD , et al. Efficacy and safety of stereotactic body radiation therapy for the treatment of pulmonary metastases from sarcoma: A potential alternative to resection. J Surg Oncol. 2016;114:65–69. 27111504 10.1002/jso.24268

[12] Chang JY , Senan S , Paul MA , et al. Stereotactic ablative radiotherapy versus lobectomy for operable stage I non‐small‐cell lung cancer: A pooled analysis of two randomised trials. Lancet Oncol. 2015;16:630–637. 25981812 10.1016/S1470-2045(15)70168-3 PMC4489408

[13] Yu W , Tang L , Lin F , et al. Stereotactic radiosurgery, a potential alternative treatment for pulmonary metastases from osteosarcoma. Int J Oncol. 2014;44:1091–1098. 24535005 10.3892/ijo.2014.2295 PMC3977803

[14] National Comprehensive Cancer Network (NCCN) . Clinical practice guidelines in oncology. Non‐small cell lung cancer. Version 7; 2017 http://www.nccn.org/professionals/physician_gls/f_guidelines.asp#nscl 10.6004/jnccn.2017.0050 28404761

[15] Nagai A , Shibamoto Y , Yoshida M , et al. Safety and efficacy of intensity‐modulated stereotactic body radiotherapy using helical tomotherapy for lung cancer and lung metastasis. Biomed Res Int. 2014;2014:473173. 24995299 10.1155/2014/473173 PMC4065754

[16] Nyman J , Hallqvist A , Lund JA , et al. SPACE: A randomized study of SBRT vs conventional fractionated radiotherapy in medically inoperable stage I NSCLC. Radiother Oncol. 2016;121:1–8. 27600155 10.1016/j.radonc.2016.08.015

